# Cardiovascular Disease in Latin American Patients with Systemic Lupus Erythematosus: A Cross-Sectional Study and a Systematic Review

**DOI:** 10.1155/2013/794383

**Published:** 2013-11-03

**Authors:** Jenny Amaya-Amaya, Juan Camilo Sarmiento-Monroy, Julián Caro-Moreno, Nicolás Molano-González, Rubén D. Mantilla, Adriana Rojas-Villarraga, Juan-Manuel Anaya

**Affiliations:** Center for Autoimmune Diseases Research (CREA), School of Medicine and Health Sciences, Universidad del Rosario, Carrera 24 No. 63C-69, 111221 Bogotá, Colombia

## Abstract

*Objective*. This study was performed to determine the prevalence of and associated risk factors for cardiovascular disease (CVD) in Latin American (LA) patients with systemic lupus erythematosus (SLE). *Methods*. First, a cross-sectional analytical study was conducted in 310 Colombian patients with SLE in whom CVD was assessed. Associated factors were examined by multivariate regression analyses. Second, a systematic review of the literature on CVD in SLE in LA was performed. *Results*. There were 133 (36.5%) Colombian SLE patients with CVD. Dyslipidemia, smoking, coffee consumption, and pleural effusion were positively associated with CVD. An independent effect of coffee consumption and cigarette on CVD was found regardless of gender and duration of disease. In the systematic review, 60 articles fulfilling the eligibility criteria were included. A wide range of CVD prevalence was found (4%–79.5%). Several studies reported ancestry, genetic factors, and polyautoimmunity as novel risk factors for such a condition. *Conclusions*. A high rate of CVD is observed in LA patients with SLE. Awareness of the observed risk factors should encourage preventive population strategies for CVD in patients with SLE aimed at facilitating the suppression of cigarette smoking and coffee consumption as well as at the tight control of dyslipidemia and other modifiable risk factors.

## 1. Introduction

Systemic lupus erythematosus (SLE) is a complex systemic autoimmune disease (AD), characterized by the production of numerous pathogenic autoantibodies [1]. Diverse heritable, hormonal, and environmental factors and immune-system aberrations contribute to the clinical expression of the disease [2]. The heterogeneous nature of SLE explains the broad spectrum of clinical manifestations (i.e., subphenotypes). SLE affects predominantly women (female-to-male ratio 9 : 1) of child-bearing age [3]. The annual incidence and prevalence range from 1.4 to 11 cases per 100,000 population, and from 7.4 to 159.4 cases per 100,000 population, respectively [4] depending on a variety of factors, including age, gender, and ancestry. African, Hispanic, and Asian ancestry significantly influence both the risk of developing the disorder and outcome [4]. A bimodal mortality was described by Urowitz et al. [5] characterized by an early peak in the first 3 years after diagnosis due to active disease, infections and glomerulonephritis, and a second peak, 4–20 years after SLE diagnosis, in which cardiovascular disease (CVD) is the main feature and cause of death. Although overall mortality for patients with SLE has improved over the past 30 years, mortality due to CVD has remained almost the same [6].

CVD is the leading cause of mortality, responsible for about 30% of deaths worldwide. Globally, 80% of total CVD deaths occur in developing countries [7]. In addition, there is strong epidemiologic evidence that CVD risk among SLE patients compared to the general population is at least doubled [8]. Noteworthy, the excessive cardiovascular (CV) events observed in SLE individuals are not fully explained by classic risk factors. Several SLE-specific factors, including disease activity and duration, and possibly specific manifestations and therapies, further increase CV risk [8]. In fact, SLE *per se* seems to be an independent risk factor for the development of accelerated atherosclerosis [9]. 

SLE is not uncommon in Latin America (LA), the geographical area defined by Mexico, Central America, South America, and the islands of the Caribbean, a rapidly growing region with almost 600 million inhabitants [10]. Latin Americans are considered a highly admixed population due to a mixed ethnicity (so called *mestizos*) that is mainly derived from a European and Amerindian inheritance [11]. The increased prevalence of chronic diseases in LA has been attributed to diverse causes including the ageing of the population and lifestyle factors such as smoking, physical inactivity, and excess alcohol intake [12]. Despite important advances in recent decades, LA remains one of the world's most unequal regions [13]. Enormous cultural differences in health perceptions in LA exist, correlating with individuals' economic and health conditions [12]. So far, some studies regarding SLE have documented differences in health status, disease prevalence, treatment outcomes, and healthcare use among different ethnic groups, suggesting that minorities influence SLE health disparities [14–19]. Thus, this study was performed to determine the prevalence and associated risk factors for CVD in Latin American patients with SLE. 

## 2. Material and Methods

### 2.1. Study Population

First, a cross-sectional analytical study was conducted in 310 Colombian patients with SLE in whom CVD was assessed. The subjects were seen at the Center for Autoimmune Diseases Research (CREA) in Bogota, Colombia. All of them fulfilled the 1997 update American College of Rheumatology classification criteria for SLE [20]. This study was done in compliance with Act 008430/1993 by Ministry of Health of the Republic of Colombia, which classified it as a minimal-risk research. The institutional review board of the Universidad del Rosario approved the study design.

Information on patient socio-demographic and cumulative clinical and laboratory data, as well as household description, were obtained by interview, standardized report form, physical examination and chart review. All data were collected in an electronic and secure database. Socio-demographic variables included age at SLE onset, disease duration, educational and socioeconomic status, current occupation, smoking habits, coffee consumption, expositional factors and physical activity. Age at onset of the disease was defined as the first subjective experience of the symptom(s) and/or sign(s) described in any of the items of the classification criteria [21]. Duration of disease was considered as the difference between age at onset and the date of first participation in the study. Educational level was recorded as the number of years of education and was divided into two groups (more or less than 9 years) of education based on the “General Law of Education” in Colombia [22, 23]. Socioeconomic status was categorized on the basis of national legislation and was divided into low (1 and 2) and high (3–6) status. Smoking habits was assessed as ever; 1–6, 6–15, and >15 packages/year; or quitter cigarette consumption. Coffee intake was asked as yes or not, and measured in cups per day (1-2, 2–4, >4). Several expositional factors were also questioned, including the use of silicone implants, hair dyes, pesticides and organic solvents [24].

### 2.2. Clinical Variables

Clinical and laboratory variables were registered as present or absent at any time during the course of the disease. Clinical features of the disease were included taking into account the revised American College of Rheumatology criteria [20] and others manifestations as follows: polyautoimmunity (coexistence of an additional AD in the same individual on the basis of international criteria) [25, 26], multiple autoimmune syndrome (presence of more than two AD in the same patient) [26–28]; familial autoimmunity, and familial autoimmune disease were also registered as the presence of any other AD and SLE in first degree relatives (FDR) respectively [27–29]. Regarding pharmacological treatment, current or past use of azathioprine, mycophenolate mofetil, cyclophosphamide, methotrexate, antimalarials (i.e., clhoroquine, hydroxychloroquine), glucocorticoids (i.e., prednisolone, metilprednisolone, and deflazacort), and biological therapy (i.e., Rituximab) were recorded.

### 2.3. Cardiovascular Assessment

Five subphenotypes were defined and assessed: first, hypertension, defined as having a blood pressure ≥140/90 mm Hg or using any antihypertensive medication [30]: systolic and diastolic blood pressures were measured twice with at least a 15-minute interval between and the averages were recorded. Second, history of stroke, third, coronary event (i.e., unstable angina, myocardial infarction (MI)), fourth, thrombotic event (other than coronary disease and carotid involvement, requiring anticoagulant treatment), fifth, carotid disease (doppler criteria or intima-media thickness ≥0.9).

### 2.4. Laboratories Measurements

Relevant laboratory variables associated with SLE were recorded. Antinuclear antibodies, antidouble strand DNA antibodies, precipitating antibodies to extractable nuclear antigens (Sm, U1-RNP, Ro/SS-A, La/SS-B), anticardiolipin IgG and IgM, antibeta 2-glycoprotein 1 IgG and IgM antibodies, and lupus anticoagulant were extracted from the patient's clinical record. Other autoantibodies including rheumatoid factor, anticyclic citrullinated peptide, -thyroperoxidase enzyme, -thyroglobulin, -Scl 70, -centromere, -mitochondrial, and -smooth muscle antibodies were also recorded. Inflammatory biomarkers, including erythrocyte sedimentation rate (ESR), and serum high sensitive C-reactive protein (CRP) levels, as well as white blood cell and platelet count, hemoglobin levels, mean corpuscular volume, coombs test, complement (i.e., C3 and C4 levels), TSH, tetraiodothyronine T4, venereal disease research laboratory, and creatinine were extracted from patient's clinical record. Likewise, serum levels of total cholesterol (TC), triglycerides (TGL), high-density lipoprotein cholesterol (HDL), low-density lipoprotein cholesterol (LDL), and glycemia were determined by blood tests provided by every patient. 

### 2.5. Assessment of Traditional Risk Factors for CVD

Patients were assessed for traditional CVD risk factors including current age (≥45 and ≥55 years for men and women, resp.) [31–34]. Type 2 diabetes mellitus (T2DM) was defined as having a fasting plasma glucose level ≥7 mmol/L (126 mg/dL), or taking any antidiabetic agents at the time of assessment [35]. A diagnosis of dyslipidemia was given if the patient had (a) hypercholesterolemia, defined as taking lipid-lowering medication as a surrogate, or having a fasting plasma TC ≥200 mg/dL (b) HDL ≤40 mg/dL, (c) hypertriglyceridemia (TGL ≥150 mg/dL), or (d) elevated LDL (≥100 mg/dL) [31, 36]. Current hemoglobin ≤12 g/dL established a diagnosis of anemia and current levels of creatinine (abnormal defined as ≥1.2 mg/dL) were evaluated as well. History of premature CAD in FDR was also assessed [37]. Patients and their past medical records were evaluated for the current or past use of aspirin or hormone replacement therapy.

### 2.6. Statistical Analysis

First, univariate analyses were done. Categorical variables were analyzed by frequencies. Kolmogorov-Smirnov normality test was done to evaluate normality for quantitative continuous variables. Parametric data are expressed as the mean and standard deviation, and nonparametric data are described as the median and interquartile range. Second, associations between traditional and nontraditional CVD risk factors were assessed by bivariate analyses. The presence of CVD was assessed through chi square tests or Fisher's exact tests when the variable was dichotomous. Parametric values were analyzed by *t* student test. Nonparametric values were analyzed by Mann-Whitney *U* test. Clinical variables with a *P* ≤ 0.25 were included in a multivariable model. Finally, a multivariate binomial logistic regression model having CVD as the dependent variable was fit. As independent factors, the model included the traditional and nontraditional associations that were statistically significant in bivariate analyses and those variables that were biologically plausible. Moreover, other logistic regression models were made, including interactions between independent factors. In both circumstances, the models were adjusted by gender and duration of the disease. Adequacies of logistic models were assessed using the Hosmer-Lemeshow goodness-of-fit test. The Nagelkerke *R* ^2^ (i.e., pseudo-*R* ^2^) was used to estimate the percentage of variance explained by the models. Adjusted odds ratios (AOR) were calculated with 95% confidence intervals (CI). The Wald statistic test was used to evaluate the significance of individual logistic regression coefficients for each independent variable. Statistical analyses were done by the Statistical Package for the Social Sciences (SPSS, v.20, Chicago, IL, USA).

### 2.7. Systematic Review of Literature

The Preferred reporting items for Systematic Reviews and Meta-analyses guidelines were followed [38] to systematically search in the following databases: PubMed, EMBASE, Scopus, SciELO, and Virtual Health Library—which includes BIREME, LILACS and many others LA sources—about CVD and SLE in LA population. Three reviewers did the search and extraction data independently (AAJC, SMJC, and CMJ) using predefined eligibility criteria, from inception up to February 2013. 

The search was done in PubMed, using the following Medical Subject Headings (MeSH terms) groups: “Lupus Erythematosus, Systemic,” “Cardiovascular Diseases;” “Latin America,” “Ethnic Groups,” “Brazil,” “Mexico,” “Colombia,” “Chile,” “Cuba,” “Panama,” “Venezuela,” “Bolivia,” “Peru,” “Argentina,” “Uruguay,” “Paraguay,” “Ecuador,” “Nicaragua,” “Surinam,” “French Guiana,” “Guatemala,” “Honduras,” “Belize,” “Costa Rica,” “El Salvador,” “Puerto Rico,” “Dominican Republic,” and “Haiti.” Each one of them was cross-referenced with the following MeSH terms/keywords: “risk factors,” “traditional risk factors,” “classic risk factors,” “nontraditional risk factors,” “novel risk factors,” “hypertension,” “metabolic syndrome,“ “obesity,” “smoking,” “tobacco,” “dyslipidemia,” “advanced age,” “menopausal status,” “family history of CVD,” “hyperhomocysteinemia,” “sedentary lifestyle,” “renal impairment,” “male gender,” “type 2 diabetes mellitus,” “insulin resistance,” “hormone replacement therapy,” “coffee,” “ancestry,” “polymorphism, genetic,” “poliautoimmunity,” “autoantibodies,” “antibodies, antiphospholipid,” “lupus coagulation inhibitor,” “circulation anticoagulants,” “antibodies, anticardiolipins,” "beta 2-glycoprotein I," “endothelial cells antibodies,” “systemic inflammation,” “c-reactive protein,” “blood sedimentation,” “tumor necrosis factor,” “cytokines,” “immune complex,” “disease activity,” “SLEDAI,” “organ damage,” “SDI,” “duration of illness,” “immune cells aberrations,” “glucocorticoids,” “steroids,” “DMARD,” “antirheumatic agents,” “methotrexate,” “biological therapy,” “rituximab,” “anti-Inflammatory agents, non-steroidal,” “azathioprine,” “vasculopathy,” “lupus nephritis,” “premature menopause,” “endogenous dyslipidemia,” “sociodemographic factors,” “hypovitaminosis D,” “vitamin D deficiency,” “low vitamin D,” “osteoporosis,” “biomarkers,” “antiphospholipid syndrome,” “thyroiditis, autoimmune,” “Graves Disease,” “Hashimoto Disease,” “scleroderma,” “Sjögren's syndrome,” “rheumatoid arthritis,” "hypertension,” “ischemic heart disease,” “coronary artery disease,” “acute coronary syndrome,” “congestive heart failure,” “myocardial infarction,” “stroke,” “angina,” “thrombosis,” “deep vein thromboses,” “pulmonary embolism,” “periphery arterial disease,” and “atherosclerosis.” Each term was cross-referenced for the greatest number of results. No limits regarding language, period of publication, or publication type were used. 

The same terms were used for searching in EMBASE and Scopus databases. Each MeSH term and keyword was translated into DeCS (Health Sciences Descriptors) in order to explore sources of information in Spanish, Portuguese, and English through SciELO and Virtual Health Library databases. 

### 2.8. Study Selection, Data Extraction, and Quality Assessment

Reviewers screened all titles and abstracts and applied the eligibility criteria in order to identify studies that were appropriate for inclusion [39]. A study was included if (a) the abstract was available, (b) it contained original data, (c) it used accepted classification criteria for SLE, (d) it measured CV risk factors (traditional and/or nontraditional), (e) it examined as a clinical endpoints: hypertension, ischemic heart disease (IHD), coronary artery disease (CAD), acute coronary syndrome (ACS), MI, angina, congestive heart failure (CHF), stroke, thrombosis, peripheral arterial disease (PAD), and subclinical atherosclerosis, and (d) it includes LA population. In order to complete the systematic review, several authors were contacted by E-mail for full text and those references from the articles that seemed to be relevant to the review were hand-searched.

Articles were excluded from the analysis if they dealt with juvenile SLE or were done on animal models (i.e., murine models) instead of SLE patients. Studies were also excluded if they were reviews or case reports, or if they discussed topics not related to CVD, and/or were not done on LA population. Blinded reviewers (AAJC, SMJC, and MJC) organized selected articles on the basis of publication source, country, author, year, type of study, sample size, traditional and nontraditional risk factors, cardiovascular outcomes evaluated, and main results. Only novel risk factors with statistical significance were included. For details, see Supplementary Table S1 in the supplementary material available online at http://dx.doi.org/10.1155/2013/794383. Each record was classified based on the quality score of the studies that was assigned by applying the levels established by the Oxford Centre for Evidence-based Medicine 2011 in order to evaluate the risk of bias [40]. The search results were compared and disagreements were resolved by consensus. 

## 3. Results

### 3.1. Description of the Study Population

Out of a total of 310 patients, 91.3% (283/310) were women. The median (interquartile range) of age and duration of the disease was 37 (22) and 5 (9) years, respectively. CVD was observed in 36.5% (113/310). The most frequent condition was hypertension (25.2%) (Tables 1 and 2).

### 3.2. Factors Associated with CVD

Current age, ever smoking, coffee consumption, polyautoimmunity (i.e., antiphospholipid syndrome), dyslipidemia, use of cytotoxic drugs, serositis, renal involvement (i.e., nephrotic syndrome), and thrombocytopenia were all risk factors significantly associated with CVD. Instead, ethnicity and leukopenia were factors negatively associated with CVD (Table 3).

### 3.3. Adjusted Effects of Risk Factors for CVD

Dyslipidemia, pleural effusion, polyautoimmunity, and renal compromise were variables significantly associated with CVD, regardless of gender and duration of the disease by logistic regression analysis (Table 4). The association between CVD and smoking habit remained statistically significant after establishing interaction with coffee consumption (Table 5).

### 3.4. Systematic Literature Review

**Figure 1:**
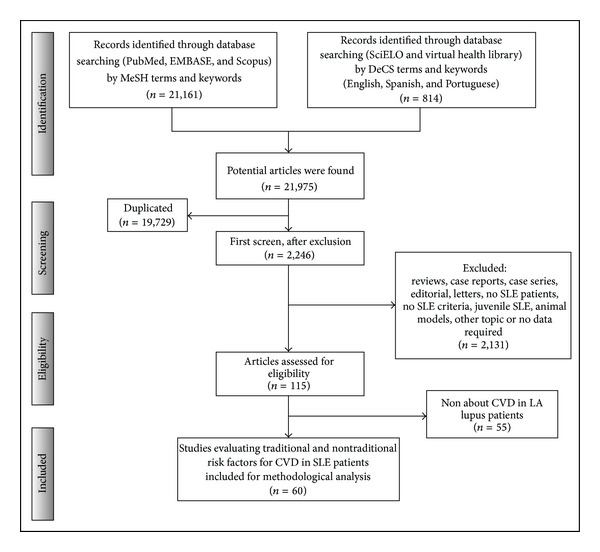
Flowchart of the systematic literature review. SLE: systemic lupus erythematosus, CVD: cardiovascular disease, LA: Latin America.

 There were 21,161 articles identified in PubMed, EMBASE, and Scopus databases search. Additional records identified through other sources included 814 articles (SciELO and Virtual Health Library). Therefore, the database searches provided a total of 21,975 publications. Of these, 19,729 were identified as duplicates. A total of 2,246 full text articles were assessed for eligibility. Only 115 articles were included for methodological analysis. Finally, 60 articles that had interpretable data and fulfilled the eligibility criteria were included. In 3 papers, data extraction was made from abstract [41–43]. For details, see Supplementary Table S1. There were 29 from Brazil [42, 44–71], 14 from Mexico [43, 72–84], 6 from United States of America [16–18, 85–87], 5 from Argentina [14, 41, 88–90], 4 from Puerto Rico [91–94], and 1 from Colombia [95] and Chile [96]. Five studies correspond to SLE LUMINA (LUpus in MInorities: nature versus nurture) multiethnic cohort [16–18, 85, 86]. The LUMINA cohort is comprised of patients of Hispanic (from Texas and Puerto Rico), African-American and Caucasian background. Two studies correspond to GLADEL'S (Grupo Latino Americano De Estudio de Lupus) longitudinal inception cohort [14, 97]. Thirty studies were cross-sectional, 15 were case-controls, 11 were cohort, 2 nested case-control and 2 were an inception cohort. The flowchart for systematic literature review and articles included in the analysis are shown in Figure 1.

### 3.5. Prevalence, Risk Factors, and Subphenotypes of CVD

**Figure 2:**
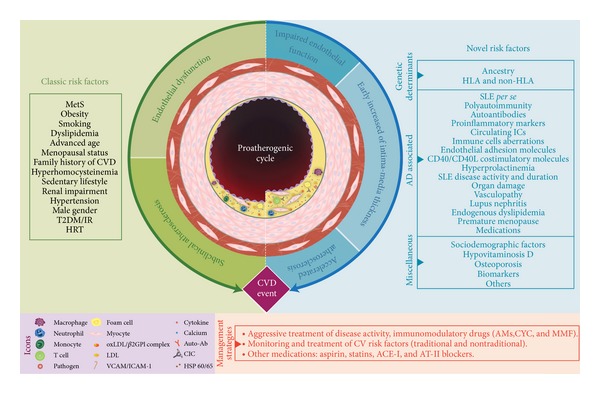
Traditional and autoimmune-related mechanisms of cardiovascular disease in systemic lupus erythematosus. A complex interaction between traditional and disease-specific traits leads to premature atherosclerotic process. Several risk factors (left) have been described since The Framingham Heart Study, known as classic risk factors, which over time conduce to endothelial dysfunction, subclinical atherosclerosis, and CV event manifest. In the autoimmune setting (right), a broad spectrum of novel risk factors contribute to development of premature vascular damage. This damage is represented by impaired endothelial function and early increased of Intima-Media Thickness which are surrogates of the accelerated atherosclerosis process, which is perpetuated by a chronic proinflammatory milieu. The cornerstone of management of CV risk include an aggressive treatment of disease activity, the continuous monitoring and treatment of modifiable CV risk factors, as well as the use of other medications in order to diminish de CV burden. CVD: cardiovascular disease, SLE: systemic lupus erythematosus, MetS: metabolic syndrome, T2DM: type 2 diabetes mellitus, IR: insulin resistance, HRT: hormone replacement therapy, CIC: Circulating Immune complex, oxLDL/B2GPI complex: oxidized-low density lipoprotein/*β*2 glycoprotein I, HDL: high density lipoprotein, Auto-Ab: auto-antibodies, AMs: antimalarials, CYC: cyclophosphamide, AZA: azathioprine, MMF: mycophenolate mofetil, ACE-I: angiotensin-converting enzyme inhibitors, AT-II blockers: angiotensin II receptor blockers.

 Out of total of 60 articles that fulfilled eligibility criteria, 46 had interpretable data regarding CVD frequency, which corresponds to a prevalence range of 4%–79.5%. Several *classic* CV risk factors such as metabolic syndrome (MetS), obesity, dyslipidemia, hypertension, T2DM, sedentary lifestyle, male gender, smoking, advanced age, hyperhomocysteinemia, renal impairment, family history of CVD, and menopausal status were described. Several studies reported *nontraditional *risk factors such as ancestry, certain single-nucleotide polymorphisms, SLE *per se*, polyautoimmunity, autoantibodies (i.e., antiphospholipid), markers of systemic inflammation (i.e., CRP), SLE disease activity, SLE duration, organ damage, immune cells aberrations, medication (i.e., glucocorticoids), vasculopathy, lupus nephritis, endogenous dyslipidemia, bone mineral density, education level, and monthly income. A broad spectrum of CV subphenotypes including hypertension, IHD, CAD, ACS, MI, angina, CHF, stroke, thrombosis, peripheral arterial disease, subclinical atherosclerosis, and mortality due to CVD were described in LA individuals with SLE. For details, see Figure 2 and Supplementary Table  S1.

## 4. Discussion

The analysis of Colombian patients with SLE discloses a high prevalence of CVD (36.5%). In contrast, prospective North Americans cohort studies showed a prevalence and annual incidence of CVD between 6 and 10%, and 1.5%, respectively [9, 98, 99]. Meanwhile, a case control study on the British General Practice Research Database showed higher risk of CVD in patients with SLE than rheumatoid arthritis [100]. Furthermore, in Italian population, Doria et al. [101] demonstrated rates of 10–40% for subclinical atherosclerosis. This inconsistency may reflect methodological obstacles such as differences in the definition of CVD outcome. Since CVD begins by endothelial dysfunction, hypertension was considered as the first subphenotype to be assessed.

### 4.1. Epidemiology of Atherosclerosis among Patients with SLE

Carotid plaque is prevalent in 21% of SLE patients under age of 35 and in up to 100% of those over age 65 [102]. The most striking example of raised risk of MI comes from the University of Pittsburgh SLE cohort, in which women with SLE aged 35–44 were >50 times more likely to experience MI than women without SLE from the Framingham Offspring study (RR 52.4; 95% CI 21.6–98.5) [8, 98].

Independent predictive risk factors for CV events had been assessed in five large prospective cohorts of patients with SLE, including Baltimore (1992, *n* = 229) [103], Pittsburg (1997, *n* = 498) [98], LUMINA (2004, *n* = 546) [17], Toronto (2007, *n* = 561) [104] and Systemic Lupus International Collaborating Clinics-Registry for Atherosclerosis (2010, *n* = 637) [105]. These cohorts found association of diverse classic risk factors (i.e., older age at diagnosis, smoking, hypercholesterolemia, male gender, and hypertension), as well as novel risk factors (i.e., longer duration of SLE and glucocorticoid use, antiphospholipid antibodies, and neuropsychiatric lupus), with CVD in SLE patients [105, 106]. In a recent meta-analysis, Schoenfeld et al. [8] showed that epidemiologic data strongly support that SLE patients are at elevated relative risk of CVD. The risks of MI, CHF, CVA, and CVD mortality are all increased among SLE patients compared to general population risks. The variability regarding the relative importance of risk factors for CVD among SLE patients in past epidemiologic studies is likely due in part to different design methods and different patient and comparison groups.

### 4.2. CVD in Hispanics with SLE

 CVD has been assessed in LUMINA multiethnic cohort and GLADEL's longitudinal inception cohort, which demonstrated differences in sociodemographic, clinical (i.e., subphenotypes), immunologic, and therapeutic characteristics, in SLE patients with CV events [14–19, 85, 86, 93]. The present study adds further evidence about the high frequency of CVD in patients with SLE, their traditional risk factors (i.e., dyslipidemia, and smoking), and highlights coffee consumption as a factor for such a complication. Through the systematic review several factors and outcomes related to CVD were also identified (Table 6). 

### 4.3. Cigarette Smoking and Coffee Consumption Independently Influence the Risk of Developing CVD

Several studies have assessed smoking as an independent risk factor for CV atherosclerotic disease [17, 107–111]. Gustafsson et al. [110] found that smoking may be the main traditional risk factor promoting increased CV risk in 208 SLE patients (RR 3.4, 95% CI 1.3–9.2). Previously, the same group found that smoking was predictive of MI, stroke, peripheral vascular disease (PVD) or CV mortality among the same patient population [109]. Toloza et al. [17] prospectively followed SLE patients over a median follow-up of 73.8 months and compared those who had a CVD event to those who did not as part of the LUMINA study. Current cigarette use was significantly associated with a 3.7-times increased risk of having a CVD event. In the PROFILE population, another multicenter, multiethnic study population, Bertoli et al. [111] found that smoking acted as an independent risk factor associated with a 2-fold decrease in time to a CV event among 1,333 SLE patients over a 6.4-year follow-up period. 

Several studies have evaluated the association between coffee consumption and CVD in the general population with controversial results. Two Dutch studies [112, 113] found no association between coffee intake, high blood pressure, and CVD. Despite the classification of coffee consumption differed among studies, some results suggest that habitual coffee consumption is associated with increased risk of hypertension [114]. In the same way, Klag et al. [115] demonstrated over many years of followup that coffee drinking is associated with small increases in blood pressure but appears to play a small role in the development of hypertension. When they compared with nondrinkers at baseline, coffee drinkers had a greater incidence of hypertension during follow-up (18.8% versus. 28.3%; *P* = 0.03). Relative risk (95% confidence interval) of hypertension associated with drinking 5 or more cups a day was 1.35 (0.87–2.08) for baseline intake and 1.60 (1.06–2.40) for intake over followup. Other effects attributed to coffee drinking are the increase in systemic vascular resistance, increased serum cholesterol levels, arterial stiffness, plasma rennin activity, epinephrine and norepinephrine, driving an unfavorable effect on endothelial function in healthy population [116]. On the other hand an Australia study [117] detected a negative association between coffee, hypertension, and MetS. Likewise, an increase in flow-mediated dilation and a decrease in CRP levels related to coffee drinking have been observed regardless of CAD [118, 119].

In order to isolate the interaction of smoking and coffee consumption, two regression models were made in which both the independent effect of coffee and smoking consumption on CVD as well as their interaction remains significant, demonstrating synergism between them (i.e., multiplicative effect). Otherwise, coffee consumption has not been evaluated systematically in SLE patients with CVD. However, since there is not a universal accepted tool for assessing coffee consumption, a bias concerning this variable is not precluded. Furthermore, membership bias could also exist because coffee consumption in Colombia is a well-defined tradition. 

### 4.4. Traditional Risk Factors for CVD in SLE

 Diverse lupus cohorts had shown the influence of advanced age, dyslipidemia, obesity, hypertension, and hyperhomocysteinemia, as classical risk factors for CVD [109, 120, 121]. Younger patients with SLE have the greatest relative risk compared to their healthy counterparts, but the absolute risk of CVD among SLE patients increases with advancing age [8]. de Souza et al. [58] observed that young Brazilian patients with SLE presented higher prevalence of carotid plaque than controls.

López-Jaramillo et al. [122] showed that the concentration of proinflammatory cytokines is higher in the LA population than in developed countries, suggesting a higher susceptibility to develop systemic low-degree inflammation at a given level of abdominal obesity, which contributes to the burden of CVD in this population. The inflammatory milieu of SLE leads to deregulation of lipid metabolism pathways, which contribute to the increased risk of atherosclerotic disease among these patients [123, 124]. Five large cohort studies have shown hypercholesterolemia as a risk factor for CVD in SLE patients [98, 103, 125–127]. Our results confirm the role of dyslipidemia as an independent risk factor for CVD in LA patients with SLE [50, 52, 53, 58, 60, 62–64, 66, 80, 81, 83, 84, 89]. For more details see Table 6.

### 4.5. Nontraditional Risk Factors for CVD in SLE

It is well known that while traditional CVD risk factors are undoubtedly important in increasing the CVD risk among SLE patients, these do not fully account for the elevated risk of CVD in this population [9]. Thereby, SLE-associated factors play an important role in the premature atherosclerosis process characteristic of these patients [128, 129]. Evidence strongly suggests that atherosclerotic plaque is largely driven by inflammation and an active immunological response [130–132] and points to SLE itself as an independent risk factor for premature CAD [133, 134]. Nontraditional risk factors for CVD in ADs may be classified into genetics, AD-related, and miscellaneous [135, 136].

Family and twin studies have repeatedly supported a role for heredity in CAD, particularly in young individuals. Several genetic markers have been proposed as predisposing factors for CVD in SLE patients [87, 137, 138]. The SLE-associated risk factors represent a broad spectrum of conditions related to the autoimmune nature of the disease. All of these pathways may eventually converge into a shared proatherogenic phenotype [139]. Table 6 summarizes the state of the art of CVD risk factors in LA patients with SLE.

### 4.6. Assessment and Management of CV Risk in SLE Patients

Physicians often face the question of how to personalize treatment and prevention of CV events. Framingham risk score (FRS) is widely used to stratify asymptomatic patients into different CV risk categories in order to target the intensity of primary medical intervention. This score is strongly influenced by age and therefore has limited usefulness in young patients with SLE [140]. Therefore, evaluation of only traditional CAD risk factors in lupus patients may result in the underestimation of their future overall CAD risk. The contribution of inflammatory biomarkers should be also considered to gain a complete picture of the CAD risk in patients with underlying conditions that increase inflammation such as SLE [141]. The Reynolds Risk score incorporates CRP concentration in the risk model and reclassifies approximately 50% of women in the 10-year FRS 5–20% risk category into different risk categories [142]. Another proposed approach corresponds to Systematic Coronary Risk Evaluation that is recommended by the EULAR experts and is widely used for CV assessment in patients with rheumatoid arthritis and other forms of inflammatory arthritis [143]. Neither of these charts includes the broad variety of risk factors that are disease-specific and could potentially explain the increased burden due to CV events. Therefore, CV risk in SLE patients is consistently underestimated with these scales.

Recently, Petri and Magder [144] proposed a data-driven risk equation of CV risk in SLE, based on data collected in a longitudinal cohort that can better estimate 10-year CV risk than the Framingham equation. In this model an integer score is given to each variable including age over 40, male gender, systolic blood pressure over 140, TC over 160, smoking, T2DM, mean SLE disease activity index (SLEDAI), history of lupus anticoagulant, and low mean C3. Using this model, together in the absence of SLE-specific risk factors, the estimated risk is higher than what would be projected based on the FRS. Nevertheless, this model is not exempt: limitations on, for example, HDL and LDL are not available at all routine visits. Further validation of this model is warranted. 

SLE entails a CVD equivalent to T2DM; thus, lower lipid goals, aspirin use, and an aggressive monitoring are required [145, 146]. Studies aimed to evaluate whether or not traditional treatment regimens prevent or slow atherosclerosis in SLE patients have been published [147]. The recent randomized controlled Lupus Atherosclerosis Prevention Study suggested that atorvastatin do not slow progression of subclinical atherosclerosis [148].

There are several new mechanisms of action described for antimalarials, many of them with beneficial effects in the management of CV risk in patients with SLE [149, 150]. There is evidence that antimalarials reduce serum cholesterol and LDL levels, elevate HDL cholesterol, and when taken concomitantly with steroids can reduce serum cholesterol [151]. Penn et al. [152] suggested that hydroxychloroquine use is associated with a lower fasting glucose and a decrease in the homeostasis model assessment-insulin resistance (HOMA-IR) index. Rekedal et al. [153] showed that hydroxychloroquine initiation was associated with a significantly greater reduction in HbA1c as compared to methotrexate initiation among diabetic patients with rheumatic diseases. In addition, beneficial effects of hydroxycloroquine on thrombosis formation have also been described. Multiple retrospective cohort studies have shown a reduced incidence of thrombotic events and improved overall survival in patients with SLE treated with antimalarials [154–157]. 

The presence of low vitamin D levels has been associated with disrupting self-tolerance. Therefore, it is tempting to speculate that vitamin D might prove useful as a preventive agent by reducing the risk of developing an autoimmune response [158]. In addition, vitamin D has been found to have antithrombotic properties improving the risk of CVD. Vitamin D deficiency has been linked to the development of atherosclerosis. As a corollary, the use of vitamin D may be useful to improve microvascular endothelial function [158].

## 5. Limitations of the Study

 As a cross-sectional study, it is placed at level 4 of evidence according to Oxford Evidence Based Medicine [40]. Although the study sample size is not negligible, it would have been more valuable to have had an appropirate followup to establish valid associations between CVD, novel risk factors, and SLE. This, in turn, could have improved both internal and external validities.

The diversity of parameters defining CVD in SLE precluded homogenization of analysis and assessment in the systematic review. Often, one or two CVD subphenotypes were assessed as primary outcomes but many did not consider general CVD compromise. Therefore, it was not possible to perform a meta-analysis and to establish true measures of association such as odds ratios. Finally, we acknowledge that publication bias may exist.

## 6. Final Remarks and Conclusions

CVD is a major cause of morbidity and mortality in SLE patients. SLE and CVD share common pathophysiology mechanisms (i.e., systemic and chronic inflammation) with secondary accelerated atherosclerosis. Since traditional risk factors do not completely explain the high rates of CVD in patients with SLE, novel risk factors related to autoimmunity are now recognized. In the current study classical risk factors such as dyslipidemia, coffee consumption, and smoking habit are highlighted, and SLE-related factors (i.e., SLE-antiphospholipid syndrome polyautoimmunity, pleural effusion, and renal involvement) are confirmed. In addition, several factors associated with CVD in LA patients with SLE were reviewed. Altogether, our results should encourage preventive population strategies for CVD in patients with SLE [106, 159], aimed at facilitating the suppression of cigarette smoking and coffee consumption as well as to the tight control of dyslipidemia and other modifiable risk factors.

## Supplementary Material

The Supplementary Material enclose the total description of the systematic literature review, organized by country, author, and year. In this table are all data regarding to type of study, evidence level and contains the prevalence found and their associations with traditional and non–traditional risk factors for CVD in patients with SLE in Latin America.

## Figures and Tables

**Table 1:** Demographic and clinical characteristics of 310 patients with SLE.

Characteristic	Median (IQR)
Age (y)	37 (22)
Duration of disease (y)	5.0 (9.0)

Characteristic	Mean (SD)

Age at SLE onset (y)	38 (15.4)
Age of diagnosis (y)	39.8 (15.8)

Sociodemographic characteristic	% (*n*/*N*)

Female	91.3 (283/310)
High education level	85.6 (255/298)
High socioeconomic status	79.5 (236/297)
Mixed occupation	23.2 (71/306)
Housewife	21.2 (65/306)
Exposure to hair dyes	40.6 (125/308)

Clinical manifestation	% (*n*/*N*)

Cutaneous compromise	88.1 (273/310)
Arthropathy	87.7 (272/310)
Neurological involvement	10 (31/310)
Hematological criteria	34.5 (107/310)
Immunological criteria	78.1 (242/310)
Raynaud's phenomenon	39.4 (122/310)
Vasculitis	18.4 (57/310)
Alopecia	47.7 (148/310)
Livedo reticularis	19 (59/310)
Pleural effusion	23.5 (73/310)
Pulmonary hypertension	6.5 (20/310)
Pulmonary embolism	2.9 (9/310)
Pericarditis	14.5 (45/310)
Lupus nephritis	46.5 (144/310)
Nephritic syndrome	5.5 (17/310)
Nephrotic syndrome	16.8 (58/310)
Histological pattern	
Normal	8.5 (7/82)
Mesangial glomerulonephritis	15.9 (13/82)
Focal segmental glomerulonephritis	13.4 (11/82)
Proliferative glomerulonephritis	41.5 (34/82)
Membranous glomerulonephritis	11 (9/82)

Autoimmune disease(s)	% (*n*/*N*)

Polyautoimmunity	26.1 (81/310)
MAS	6.1 (19/310)
Familial autoimmunity in FDR	30.3 (94/310)
RA	3.9 (12/310)
APS	8.7 (27/310)
SS	8.7 (27/310)
AITD	6.8 (21/310)

Comorbidities	% (*n*/*N*)

Fibromyalgia	9.7 (30/309)
Depression	21.4 (66/309)
Epilepsy	3.9 (12/309)
Peptic ulcer disease	38.5 (119/309)
Anemia	8.1 (25/309)
Osteoporosis	5.8 (18/309)
Malaria	2.3 (7/303)
Hepatitis A	7.1 (22/309)
Miscarriage	20.2 (57/282)

Drugs	% (*n*/*N*)

Azathioprine	33.9 (105/310)
Antimalarial	80.6 (250/310)
Mycophenolate Mofetil	15.8 (49/310)
Steroid	78.4 (243/310)
Rituximab	7.4 (23/310)
Cytotoxic agents	12.3 (38/310)
Biological treatment	8.4 (26/310)
Methotrexate	34.8 (108/310)

Laboratories findings	% (*n*/*N*)

Anemia	25.9 (76/293)
Leukopenia	38 (114/300)
Lymphopenia	78.7 (85/202)
Thrombocytopenia	7.8 (23/296)
C-reactive protein (+)	35.4 (57/161)
Erythrocyte sedimentation rate (+)	46.2 (104/225)
VDRL (+)	24.5 (26/204)
Abnormal serum creatinine	9.7 (26/268)
Abnormal creatinine clearance	56.6 (94/166)
24 hours proteinuria (+)	37.7 (80/212)
Hematuria	57.4 (58/101)
Pyuria	36.1 (56/155)
Antinuclear antibodies (+)	98.6 (287/291)
Lupus anticoagulant (+)	51 (52/102)
Anti-dsDNA antibodies (+)	54.6 (147/269)
Low complement 3 (C3)	60.8 (160/263)
Low complement 4 (C4)	31.4 (83/264)
aCL IgG (+)	33.8 (79/234)
aCL IgM (+)	32.4 (73/225)
Beta 2-glycoprotein antibodies IgG (+)	29.4 (10/34)
Beta 2-glycoprotein antibodies IgM (+)	28.6 (6/21)
Anti-Ro antibodies (+)	48.6 (122/251)
Anti-La antibodies (+)	26.2 (64/244)
Anti-Sm antibodies (+)	36 (89/247)
Anti-RNP antibodies (+)	46 (109/237)
Rheumatoid factor (+)	34.6 (27/78)
Citrullinated peptide antibodies (+)	34.3 (12/35)
Thyroid stimulating hormone (+)	51.5 (53/207)
Thyroid peroxidase antibodies (+)	38.6 (17/44)
Thyroglobulin antibodies (+)	20 (6/30)

CVD: cardiovascular disease; SLE: systemic lupus erythematosus; IQR: interquartile range; SD: standard deviation; Y: years; MAS: multiple autoimmune syndrome; FDR: first-degree relatives; RA: rheumatoid arthritis; APS: antiphospholipid syndrome; SS: Sjögren's syndrome; AITD: autoimmune thyroid disease; VDRL: venereal disease research laboratory; aCL: anticardiolipin antibodies.

Arthropathy was defined as the presence of at least one of the following: arthritis, arthralgia, hands edema, or Jaccoud's arthropathy.

Cutaneous compromise was defined as the presence of at least one of the following: photosensitivity, oral ulcers, malar rash, discoid lupus, subacute lupus, and urticaria.

Neurological involvement were defined as the presence of at least one of the following: seizures, psychosis, and peripheral nerve compromise.

Hematological criteria were defined as the presence of at least one of the following: hemolytic anemia, leukopenia, lymphopenia, and thrombocytopenia.

Immunological criteria were defined as the presence of at least one of the following: anti-dsDNA, anti-Sm, anticardiolipin IgG or IgM, lupus anticoagulant, false-positive test VDRL, or fluorescent treponemal antibody absorption test.

**Table 2:** Cardiovascular disease characteristics of 310 patients with SLE.

	% (*n*/*N*)
*Clinical manifestation *	
Cardiovascular disease	36.5 (113/310)
Hypertension	25.2 (78/310)
Stroke	16.8 (52/310)
Coronary disease	2.6 (8/310)
Thrombotic event	1.6 (5/310)
Carotid disease	0.6 (2/310)

*Risk factors *	
Type 2 diabetes mellitus	1.9 (6/309)
Dyslipidemia	18.1 (56/309)
Ever smoking	39.21 (120/306)
1 to 6 packages/year	4.2 (13/306)
6 to 15 packages/year	2.6 (8/306)
More than 15 packages/year	2.3 (7/306)
Quitter	30.1 (92/306)
Coffee consumption	61.5 (187/304)
1 to 2 cups/day	30.9 (94/304)
2 to 4 cups/day	22.7 (69/304)
More than 4 cups/day	7.9 (24/304)
Never	38.5 (117/304)

*Laboratories findings *	
Abnormal triglycerides	34.3 (12/35)
Abnormal total cholesterol	44.4 (16/22)
Abnormal high-density cholesterol	58.8 (20/34)
Abnormal low-density cholesterol	33.3 (8/24)
Abnormal glycemic	25.7 (9/35)

**Table 3:** Characteristics associated with CVD in 310 patients with SLE.

Characteristic	Cardiovascular	Noncardiovascular	OR (95% CI)	*P*
	disease 113/310	disease 197/310		
	% (*n*/*N*)	% (*n*/*N*)		
Sociodemographic characteristic				
Age (y)	Median (IQR): 40 (23)	Median (IQR): 36 (21)		0.059
Age at SLE onset (y)	Median (IQR): 30 (19)	Median (IQR): 26 (18)		0.175
Age of diagnosis (y)	Median (IQR): 34 (22)	Median (IQR): 28 (18)		0.041
Ever smoking	50.9 (57/112)	37.1 (73/194)	1.75 (1.09–2.61)	0.019
Coffee	70.5 (79/112)	55.2 (108/192)	1.94 (1.19–3.18)	0.009
Hair dye	46 (52/113)	37.4 (73/195)	1.42 (0.89–2.29)	0.139
Pesticides	3.5 (4/113)	1 (2/197)	3.56 (0.64–19.75)	0.122
Autoimmune disease(s)				
MAS	3.5 (4/113)	7.1 (14/197)	0.48 (0.15–1.49)	0.196
Familial autoimmunity in FDR	19.5 (22/113)	25.4 (50/197)	0.71 (0.40–1.25)	0.235
RA	0.9 (1/113)	5.6 (11/197)	0.15 (0.01–1.19)	0.062
APS	16.8 (19/113)	4.1 (8/197)	4.77 (2.01–11.31)	0.0001
AITD	4.4 (5/113)	9.1 (16/197)	0.52 (0.18–1.47)	0.213
Comorbidities				
Type 2 diabetes mellitus	3.5 (4/113)	1 (2/197)	3.56 (0.64–19.75)	0.196
Dyslipidemia	28.3 (32/113)	12.2 (24/197)	2.83 (1.56–5.11)	0.0001
Fibromyalgia	14.2 (16/113)	7.1 (14/197)	2.14 (1.00–4.57)	0.045
Treatment				
Antimalarials	77 (87/113)	82.7 (163/197)	0.69 (0.39–1.23)	0.217
Mycophenolate Mofetil	19.5 (22/113)	13.7 (27/197)	1.52 (0.82–2.82)	0.181
Cytotoxics agents	17.7 (20/113)	9.1 (16/197)	2.13 (1.07–4.23)	0.027
Clinical variable				
Discoid lupus	5.3 (6/113)	9.1 (16/197)	0.55 (0.21–1.44)	0.225
Alopecia	41.5 (47/113)	51.3 (101/197)	0.67 (0.42–1.08)	0.101
Subacute	6.2 (7/113)	3 (6/197)	2.10 (0.68–6.41)	0.239
Urticaria	19.5 (22)	10.2 (20/197)	2.14 (1.11–4.12)	0.021
Vasculitis	15 (17/113)	20.3 (40/197)	0.89 (0.37–1.29)	0.250
Neurological involvement	13.3 (15/113)	8.1 (16/197)	1.73 (0.82–3.65)	0.146
Headache	28.2 (33/113)	18.9 (37/197)	1.79 (1.03–3.06)	0.035
Psychosis	7.1 (8/113)	3.8 (7/197)	2.08 (0.72–5.86)	0.164
Serositis	37.2 (42/113)	23.9 (47/197)	1.88 (1.14–3.12)	0.013
Pleural effusion	31.9 (36/113)	18.9 (37/197)	2.02 (1.18–3.44)	0.009
Hands edema	32.7 (37/113)	15.9 (33/197)	2.41 (1.40–4.16)	0.0001
Renal involvement	56.6 (84/113)	40.9 (80/197)	1.91 (1.19–3.05)	0.006
Nephrotic	27.4 (31/113)	10.7 (21/197)	3.16 (1.71–5.84)	0.0001
Pulmonary haemorrhage	3.5 (4/113)	0.5 (1/197)	7.19 (0.79–65.16)	0.061
Laboratory findings				
Thrombocytopenia	11.9 (13/109)	5.3 (10/187)	2.39 (1.01–5.67)	0.041
Leukopenia	30.6 (34/111)	42.3 (80/189)	0.60 (0.36–0.98)	0.044
Lymphopenia	71.6 (78/109)	78.7 (148/188)	0.68 (0.39–1.17)	0.163
Abnormal creatinine	17 (17/100)	5.4 (8/169)	3.61 (1.54–8.47)	0.002
Abnormal creatinine clearance	64.4 (38/59)	52.3 (56/107)	1.64 (0.85–3.17)	0.133
Proteinuria (+)	50 (41/82)	30 (39/130)	2.33 (1.31–4.13)	0.003
aCLIgG (+)	40.7 (35/88)	29.7 (44/149)	1.62 (0.93–2.82)	0.087
Lupus anticoagulant (+)	58.5 (24/41)	45.9 (29/61)	1.66 (0.74–3.70)	0.211

CVD: cardiovascular disease; SLE: systemic lupus erythematosus; OR: odds ratio; CI: confidence interval; IQR: interquartile range; SD: standard deviation; Y: years; MAS: multiple autoimmune syndrome; FDR: first-degree relatives; RA: rheumatoid arthritis; APS: antiphospholipid syndrome; AITD: autoimmune thyroid disease; aCL: anticardiolipin antibodies.

**Table 4:** Factors associated with CVD in patients with SLE*.

Characteristic	*β*	AOR	95% CI	*P*
Dyslipidemia	0.971	2.64	1.32–5.28	0.005
Pleural effusion	0.751	2.12	1.17–3.84	0.013
Ever smoking	0.602	1.83	1.07–3.10	0.025
Coffee consumption	0.559	1.75	1.01–3.04	0.043
Renal involvement	0.476	1.61	0.94–3.84	0.081

CVD: cardiovascular disease; SLE: systemic lupus erythematosus; *β*: *β* coefficient; AOR: adjusted odds ratio; 95% CI: 95% confidence interval.

*The model was adjusted by gender and duration of the disease.

**Table 5:** Factors associated with CVD in patients with SLE including interaction between smoking and coffee consumption*.

Characteristic	*β*	AOR	95% CI	*P*
APS	1.55	4.71	1.81–12.2	0.001
Dyslipidemia	1.07	2.92	1.54–5.55	0.001
Pleural effusion	0.78	2.19	1.20–3.98	0.011
Smoking and coffee	0.60	1.82	1.05–3.13	0.03

CVD: cardiovascular disease; SLE: systemic lupus erythematosus; *β*: *β* coefficient; AOR: adjusted odds ratio; CI: confidence interval; APS: antiphospholipid syndrome.

*Adjusted by gender and duration of the disease including interaction between smoking and coffee consumption. *P*-values persisted significant despite the evaluation of the four possible combinations (i.e., smoking, coffee, smoking and coffee, none) through the adjustment of the multivariate model.

**Table 6:** Traditional and nontraditional risk factors associated with cardiovascular disease and systemic lupus erythematosus in Latin America.

Risk factor associated with CVD	Comments	Reference(s)
Traditional		
Hypertension	Hypertension influences the risk of death by CVD in SLE patients.	[64, 89]
	Hypertension acts as CVD subphenotype as well as a risk factor.	[45, 49, 71, 88]
	Patients with SLE were at increased risk of thrombosis when it is associated with hypertension.	[17, 80, 84]
	Compared with patients without atherosclerotic plaque, those with plaque had higher prevalence of hypertension.	[54, 63, 66, 73]
	Lupus patients with abnormal myocardial scintigraphic findings and hypertension, as a risk factor for CAD, had a higher risk of abnormal findings on coronary angiography.	[52, 53]
	Patients with lupus had higher hypertension prevalence than controls with noninflammatory disorders.	[14, 68, 90]
T2DM	T2DM influence on abnormal myocardial perfusion in asymptomatic patients with SLE.	[53]
	Alterations in glycemic profile were associated with traditional risk factors for CHD and lupus characteristics, including CVD, damage index, and renal involvement.	[17, 68, 81]
	Patients with SLE and T2DM were at increased risk of thrombosis. This risk remains elevated throughout the course of the disease.	[16, 80]
	T2DM is an independent risk factor for atherosclerotic plaque and CAC.	[63, 71, 84]
Dyslipidemia	The main risk factor for death in SLE was heart involvement, which was influenced by dyslipidemia.	[50, 89]
	High levels of TGL were associated with myocardial perfusion abnormalities and endothelial dysfunction	[52, 53, 83]
	There was high prevalence of dyslipidemia as risk factor for thrombotic events.	[60, 62, 80]
	Alterations in lipid profile was a risk factor for premature CAC in young women with SLE.	[66, 84]
	CAD was more prevalent in dyslipidemic women with SLE than controls.	[64, 81]
	Compared with patients without atherosclerotic plaque, those with plaque had high level of TGL and LDL.	[58, 63]
Male gender	Male gender was a risk factor for developing severe organ damage (CVD) and mortality in SLE patients.	[16, 17, 53, 60]
	Males with SLE were at increased risk of thrombosis and CAC. This risk remains elevated throughout the course of the disease.	[80, 84, 85]
	Patients had more peripheral vascular and gonadal involvement compared with published data from non-Hispanic SLE populations.	[76]
MetS	SLE patients had a high prevalence of MetS that directly contributes to increasing inflammatory status and oxidative stress.	[69]
	MetS was associated with traditional risk factors for CHD and lupus characteristics, including CVD, damage Index, and renal involvement.	[68, 81]
	Presence of MetS was related to CVD in SLE patients.	[90, 94]
Obesity	Patients with SLE who had excess weight present distinct clinical-laboratory findings, sociodemographic characteristics, and treatment options when compared to normal weight patients.	[17, 71, 81]
	Excess weight is associated with some traditional risk factors for CVD and SLE poor prognosis.	[58, 65, 68]
	Increase weight influence on abnormal myocardial perfusion in asymptomatic patients with SLE.	[53, 64]
	SLE patients with high BMI have increased QT interval parameters when compared to controls. This prolongation may lead to an increased CV risk.	[55]
	Major values in BMI were related with the presence of CAD and carotid plaque.	[58, 63, 64]
Smoking	Smoking is an important determinant in the occurrence of thrombotic (central and/or peripheral, arterial and/or venous) events in SLE patients.	[18, 69, 81]
	Smoking was an independent risk factor for atherosclerotic plaque and thrombosis.	[63, 68, 80]
	Smoking habit influence on abnormal myocardial perfusion in asymptomatic patients with SLE.	[53]
	Smoking was a risk factor for premature CAC in young women with SLE.	[66, 84]
	CAD was more prevalent in women with SLE.	[64, 85, 86]
Advance age	Several traditional risk factors, including age, appear to be important contributors to atherosclerotic CV damage.	[16, 71]
	The presence of CVD has been associated with older age.	[16, 59]
	Age was directly related with atherosclerotic plaque formation.	[63]
Menopausal status	High percentage of SLE patients with abnormal angiographic findings was in postmenopausal status.	[52]
	There is high prevalence of premature menopausal status as a risk factor for CVD.	[60]
	Postmenopausal status was a risk factor for premature CAC in young women with SLE.	[66, 68, 84]
	Postmenopausal women had a higher prevalence of subclinical AT and abnormal myocardial perfusion in asymptomatic patients with SLE.	[53, 63]
Family history of CVD	Familial history of CVD was an independent risk factor for atherosclerotic process.	[17, 63, 68]
	Family history of CVD was a risk factor for premature CAC in young women with SLE.	[66, 84]
	Family history of CVD influence on abnormal myocardial perfusion in asymptomatic patients with SLE.	[53]
HRT	HRT use was not associated with the occurrence of vascular arterial events in the LUMINA patients. HRT use in women with SLE should be individualized, but data suggest its use may be safe if aPL antibodies are not present or vascular arterial events have not previously occurred.	[17]
Hyperhomocysteinemia	Hyperhomocysteinemia was a risk factor for CAC in SLE patients.	[84]
	The presence of polyautoimmunity and hyperhomocysteinemia was risk factors for thrombotic events.	[41]

Nontraditional		
*Genetic determinants *		
Ancestry	There are several differences regarding clinical (including CVD), prognostic, socioeconomic, educational, and access to medical care features in GLADEL cohort according to ancestry (White, Mestizo, and African-LA).	[14]
Non-HLA	An SNP in FGG rs2066865 demonstrated association with arterial thrombosis risk in Hispanic Americans patients with SLE.	[87]
	The CRP GT20 variant is more likely to occur in African-American and Hispanic SLE patients than in Caucasian ones, and SLE patients carrying the GT20 allele are more likely to develop vascular arterial events (LUMINA multiethnic cohort).	[86]
*SLE-associated *		
Poliautoimmunity	The presence of APS was the major independent contributor to the development of severe organ damage in Brazilian patients with SLE.	[54]
	APS and its characteristic antibodies may contribute to the development of thrombotic events in Brazilian and Mexican lupus patients.	[57, 78]
	APS had high impact in CVD and survival in Brazilian lupus patients.	[42]
	Polyautoimmunity (APS) may suggest concerted pathogenic actions with other autoantibodies in the development of thrombotic events in Mexican patients with SLE.	[78]
SLE *per se *	SLE diagnosis was significantly associated with carotid plaque formation and development of CV event in Brazilian patients with SLE.	[58]
	High percentage of patients with abnormal angiographic findings had higher ACR criteria number for SLE Brazilian patients with SLE.	[52]
Autoantibodies	One of the independent predictors of vascular events in a multiethnic US cohort (LUMINA) was the presence of any aPL antibody.	[17]
	anti-*β*2GPI antibodies were strongly associated with thrombosis in patients with Mexicans with SLE. The decrease of anti-*β*2GPI levels at the time of thrombosis may indicate a pathogenic role.	[77]
	The higher frequency of aPT found in Mexican patients with SLE with thrombosis may suggest concerted pathogenic actions with other autoantibodies in the development of thrombotic events.	[78]
	Patients with aCL antibodies seem to be at an increased risk for arterial and venous thrombotic events in Puerto Ricans and Chilean patients with SLE.	[92, 96]
	There was correlation between lupus anticoagulant and thrombotic events in Brazilian lupus patients.	[50]
	aCL antibodies were associated with thrombotic events, mainly in high titers in Chilean SLE patients.	[96]
	aCL antibodies were significantly associated with CV events and showed an association with echocardiographic abnormalities in Brazilian patients with SLE.	[51]
	Mexican patients had more peripheral vascular compared with published data from non-Hispanic SLE populations.	[76]
Immune cells aberrations	Complement fixing activity of aCL antibodies seems to be relevant in thrombotic venous events in Brazilian patients with SLE.	[57]
Inflammatory markers	Increased ESR was independently associated with MetS in Puerto Ricans lupic patients.	[94]
	One of the independent predictors of vascular events in a multiethnic US cohort (LUMINA) was elevated serum levels of CRP.	[16, 17]
Endogenous dyslipidemia	HDL distribution and composition (−HDL2b, +HDL3b, and +HDL3c) were abnormal in noncomplicated Mexican SLE patients.	[79]
	Low HDL levels and increased TGL levels were associated with atherosclerosis by cIMT measurement in Colombian lupic patients.	[95]
	SLE patients have a lipid profile abnormality in Brazilian patients with SLE. This pattern of dyslipoproteinemia may increase the risk of developing CAD.	[47]
Disease activity	Disease activity (SLAM) is an important determinant in the occurrence of thrombotic (central and/or peripheral, arterial, and/or venous) events in the LUMINA cohort.	[18]
	SLEDAI scores were positively correlated with BMI and WC in Brazilian population with SLE.	[69]
	Higher disease activity was independently associated with MetS and thrombosis in Puerto Ricans and Mexican SLE patients.	[80, 94]
	Higher score of SLICC was associated with atherosclerotic plaque in Brazilian SLE patients.	[58]
	High scores in diseases activity index (SLEDAI and SLICC) were associated with myocardial perfusion abnormalities in Brazilian SLE patients.	[52]
	Brazilian SLE patients have a lipid profile abnormality which is aggravated by disease activity and may reside in a defect of VLDL metabolism.	[47]
	Disease activity was predictor of CAC in Mexican SLE patients.	[84]
	Higher disease activity was independently associated with MetS in Puerto Ricans patients with SLE.	[94]
Organ damage	Baseline and accrued damage increase mortality risk (including due to CVD) in Brazilian patients with SLE.	[61]
	Mexican patients had more peripheral vascular involvement (measured by SDI), compared with published data from non-Hispanic SLE populations.	[76]
	In Brazilian SLE patients, MetS was associated with both traditional risk factors for CHD and lupus characteristics including damage index.	[68]
	There was a correlation between IMT and revised damage index (SLICC) in Brazilian SLE patients.	[58]
	Atherosclerotic CV damage in SLE is multifactorial, and disease-related factors (including CRP levels and SDI at baseline) appear to be important contributors to such an occurrence (LUMINA multiethnic cohort).	[16]
Long duration	Longer duration of SLE was associated with atherosclerotic plaque and CV events in Brazilian population.	[58, 59]
	A correlation between IMT and duration of the disease was found in Brazilian patients with SLE.	[63]
	Disease duration was independent predictor for premature CAC in young Brazilian women with SLE.	[66]
Medications	PDN > 10 mg/day was independently associated with MetS in Puerto Ricans SLE patients.	[94]
	In Brazilian SLE patients, there was a correlation between IMT and the duration of PDN use.	[63]
	IHD was observed in two types of Mexican SLE patients: those with long-term steroid therapy and those with frank episodes of vasculitis.	[73]
Vasculopathy	Current vasculitis was associated with abnormal myocardial scintigraphy in Brazilian patients with SLE.	[53]
	Puerto Ricans patients with SLE and RP seem to be at increased risk for arterial and venous thrombotic events.	[92]
	IHD was observed in two types of Mexican SLE patients: those with long-term steroid therapy and those with frank episodes of vasculitis.	[73]
Renal involvement	In Brazilian SLE patients, MetS was associated with traditional risk factors for CHD and lupus characteristics, including damage index and renal involvement (nephritic syndrome).	[68]
*Miscellaneous *		
BMD	Decreased BMD was an independent predictor for premature CAC in Brazilian young women with SLE.	[66]
Sociodemographic factors	A low education and monthly income were associated with MetS in Mexican patients with SLE and RA.	[81]

aCL: anticardiolipins antibodies; ACR: American College of Rheumatology; anti-*β*2GPI: anti-beta2 glycoprotein 1 antibodies; aPT: antiprothrombin antibodies; aPL: antiphospholipid antibodies; APS: antiphospholipid syndrome; AT: atherosclerosis; BMD: bone mineral density; BMI: body mass index; CAC: coronary artery calcification; CAD: coronary artery disease; cIMT: carotid Intimal Medial Thickness; CHD: coronary heart disease; CRP: C-reactive protein; CV: cardiovascular; CVD: cardiovascular disease; ESR: erythrocyte sedimentation rate; GLADEL: Grupo Latino Americano De Estudio de Lupus; HDL: high-density lipoprotein cholesterol; HRT: hormone replacement therapy; IHD: ischemic heart disease; IMT: intimal media thickness; LA: Latin America; LDL: low-density lipoprotein cholesterol; LUMINA: LUpus in MInorities: NAture versus nurture cohort; MetS: metabolic syndrome; PDN: prednisolone; RP: Raynaud's phenomenon; T2DM: type 2 diabetes mellitus; TGL: triglycerides; SLAM: Systemic Lupus Activity Measure; SLE: systemic lupus erythematosus; SLEDAI: systemic lupus erythematosus disease activity index; SLICC: Systemic Lupus International Collaborating Clinics score; SDI: SLICC damage index; SNP: single-nucleotide polymorphism; VLDL: very low-density lipoprotein cholesterol; WC: waist circumference.
